# Evaluation of the environ SRS immobilization mask system for HyperArc: Dosimetric attenuation, dose calculation accuracy, and clinical setup reproducibility

**DOI:** 10.1002/acm2.70767

**Published:** 2026-08-28

**Authors:** Adi Robinson, Matthew R. Caldwell

**Affiliations:** ^1^ Department of Radiation Oncology AdventHealth Celebration Celebration Florida USA

**Keywords:** AcurosXB, HyperArc, immobilization mask, stereotactic radiosurgery

## Abstract

**Background:**

Frameless thermoplastic masks are standard for intracranial stereotactic radiosurgery (SRS), with the Encompass (CQ Medical) widely used on the Varian HyperArc platform. A candidate mask must minimize beam attenuation, permit accurate dose calculation, and enable reproducible setup, yet these are rarely assessed together. Comprehensive comparative data for the proposed Environ (MacroMedics) mask are limited.

**Purpose:**

To compare the Environ and Encompass masks across three domains: beam attenuation, dose‐calculation accuracy under different algorithm and mask‐contouring conditions, and interfraction setup reproducibility.

**Methods:**

Attenuation was measured with an ionization chamber in a head phantom (TrueBeam, 6 FFF) at seven static gantry angles and a 0°–180° arc, with an estimated measurement uncertainty budget. Planning accuracy was assessed using HyperArc plans on phantom CT for 1–15 mm PTV margins (single‐target) and 3/5/7 mm (multi‐metastasis), calculated in Eclipse with AAA and Acuros XB (AXB), each with and without the mask in the body contour. Setup reproducibility was analyzed retrospectively from CBCT six‐degree‐of‐freedom corrections (Encompass: 67 fractions/21 patients; Environ: 72 fractions/24 patients). Because repeated fractions are not independent, positioning data were analyzed at the patient level (Mann–Whitney *U*, Levene's test), by within‐patient reproducibility, and with a confirmatory mixed‐effects model.

**Results:**

The Encompass attenuated 1.13% ± 0.59% more than the Environ for static beams (*p* = 0.002); the arc difference (0.06%) was within uncertainty (∼0.4%, *k* = 1). The Encompass required mask contouring with AXB for acceptable agreement (2.38% without), whereas the Environ achieved sub‐1% AXB agreement without contouring (0.24%). The Environ showed a smaller mean vertical correction (mixed‐effects *p* = 0.016) and reduced within‐patient variability in vertical (0.31 vs. 1.25 mm, *p* < 0.001) and pitch (0.50 vs. 0.99°, *p* = 0.009); other axes were comparable.

**Conclusion:**

The Environ performed comparably or favorably across all three domains, supporting it as a clinically acceptable SRS alternative. It achieved acceptable AXB agreement without mask contouring under the conditions tested, though this depends on institution‐specific settings and should be verified locally. The setup‐reproducibility advantages, most robust vertically, are hypothesis‐generating given the retrospective design and absence of intrafraction data.

## 1 INTRODUCTION

Stereotactic radiosurgery (SRS) delivers highly conformal, ablative radiation doses to intracranial targets in one or a few fractions. The precision demands of SRS, typically requiring submillimeter targeting accuracy, make immobilization a critical component of the treatment chain.^1^, ^2^ Historically, invasive frame‐based fixation provided the gold standard for SRS immobilization, but the adoption of frameless mask‐based systems has expanded rapidly due to their noninvasive nature, patient comfort, and compatibility with fractionated stereotactic treatments.^3^, ^4^

Modern frameless SRS immobilization systems must satisfy multiple performance criteria simultaneously. They must provide sufficient mechanical restraint to limit patient motion during treatment, maintain reproducible positioning across fractions, and introduce minimal dosimetric perturbation to the treatment beams.^5^ This last criterion has received increasing attention as treatment techniques have evolved toward highly modulated, non‐coplanar arc deliveries such as Varian HyperArc, where multiple beams traverse the mask material at varying angles and thicknesses.^6^, ^7^

The Encompass mask (CQ Medical, Avondale, PA) is a widely adopted open‐face thermoplastic immobilization system designed for SRS applications and is currently the standard mask used with the HyperArc platform. Snyder et al. evaluated the dosimetric properties of this system and demonstrated significant attenuation at the mask insert interface, with up to 17% attenuation measured for beams passing through the attachment region.^8^ The Environ mask (MacroMedics, Moordrecht, Netherlands) represents a new generation immobilization system that has been proposed as an alternative, with potential advantages in material composition and design. Before clinical adoption of a new immobilization device, a thorough evaluation of its dosimetric and positional performance relative to the established system is essential.

Previous studies have investigated individual aspects of SRS mask performance, including beam attenuation measurements^5^, ^8^ and positional reproducibility.^3^, ^4^, ^9^ However, comprehensive evaluations that simultaneously address absorption characteristics, treatment planning accuracy under different contouring and calculation conditions, and clinical positioning data are limited. Furthermore, the impact of including or excluding the mask in the body contour for dose calculation, and the interaction of this choice with the dose calculation algorithm (Analytical Anisotropic Algorithm [AAA] vs. Acuros XB [AXB]), has not been systematically reported for these specific mask systems. Recent work has further clarified the role of immobilization in modern cranial SRS: surface‐guided radiotherapy is increasingly used for setup and continuous intrafraction monitoring with open‐face masks,^10^ and analyses of multi‐target single‐isocenter treatments have derived submillimeter PTV margins from measured intrafraction motion.^11^ These developments reinforce the distinction between interfraction setup reproducibility and intrafraction immobilization performance that frames the present positioning analysis. Studies have demonstrated that the AXB algorithm more accurately handles heterogeneity corrections than AAA, with particular relevance at material interfaces.^12^, ^13^, ^14^, ^15^

The purpose of this study was to perform a three‐part evaluation of the Environ mask system relative to the Encompass mask: (1) dosimetric absorption comparison using phantom‐based ion chamber measurements, (2) treatment planning accuracy assessment comparing calculation algorithms and mask contouring approaches using HyperArc plans, and (3) retrospective analysis of clinical patient positioning data.

## 2 MATERIALS AND METHODS

### 2.1 Dosimetric absorption comparison

**FIGURE 1:**
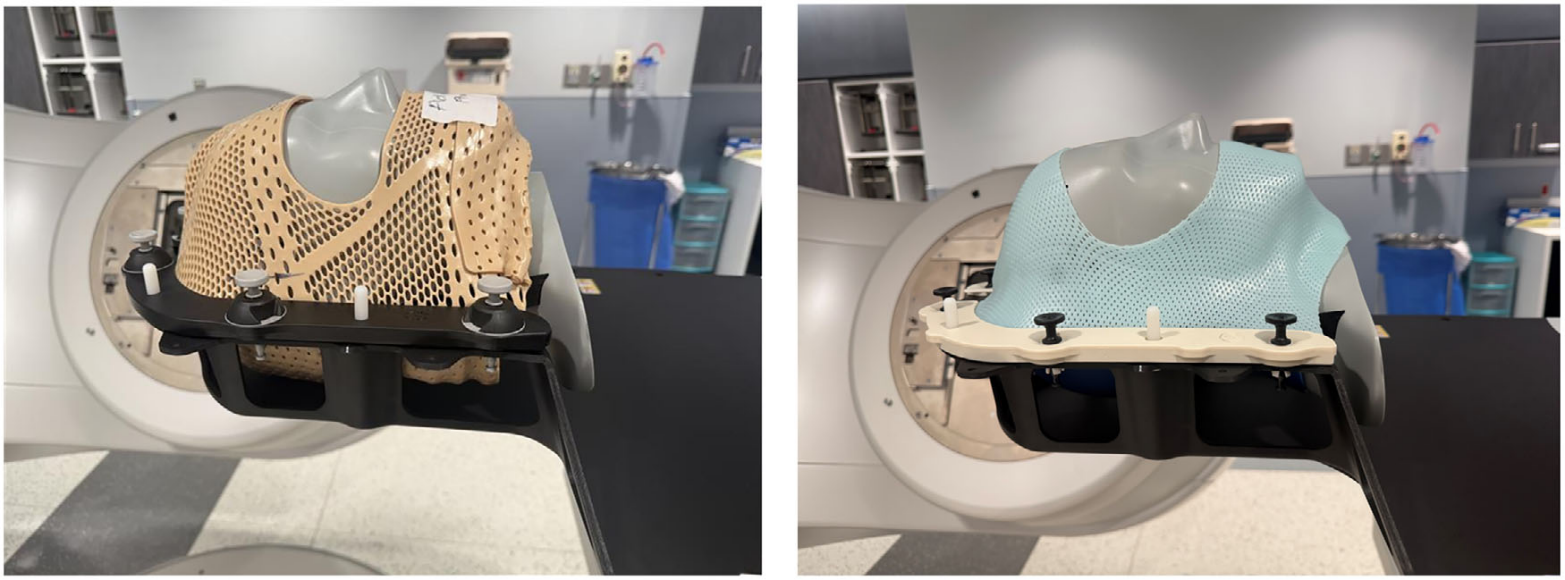
Experimental setup showing the head phantom immobilized with (left) the Encompass mask system and (right) the Environ mask system. The ionization chamber is positioned at isocenter within the phantom for both configurations.

A head phantom was immobilized using each mask system in turn (Figure 1), and a calibrated small‐volume ionization chamber (0.125 cc) was positioned at isocenter inside the phantom. All measurements were performed on a Varian TrueBeam linear accelerator equipped with a Millennium 120 multileaf collimator (MLC) using 6 MV flattening‐filter‐free (6 FFF) photon beams. The measurement methodology follows the general framework recommended by AAPM Task Group 176 for evaluating dosimetric effects of immobilization devices.^5^

Two delivery configurations were used. For the static gantry plan, seven individual beams were delivered at gantry angles of 0°, 30°, 60°, 90°, 120°, 150°, and 180°, each with a 5 × 5 cm^2^ field size and 50 monitor units (MU). Duplicate readings were acquired for each beam at each angle. For the arc plan, a single arc spanning gantry angles 0°–180° was delivered with a 5 × 5 cm^2^ field and 400 MU, also with duplicate readings.

The ion chamber was cross calibrated on the day of measurement by delivering a known dose under reference conditions (6 FFF, 10 × 10 cm^2^ field, 10 cm depth), and a charge‐to‐dose calibration factor was obtained. Temperature and pressure corrections were monitored at the beginning and end of each measurement session to confirm stability. Beam attenuation was quantified as the relative percent difference between mask systems for each measurement configuration. Statistical comparison of the static gantry measurements was performed using a paired *t*‐test, with significance defined at *p* < 0.05.

An uncertainty analysis was performed to contextualize the magnitude of the measured attenuation differences. Each beam configuration was measured in duplicate; agreement between repeat readings was within 0.2% for all configurations, corresponding to a Type A (repeatability) standard uncertainty of approximately 0.1%. Additional Type B contributions were estimated for chamber positioning reproducibility (≈0.3%), linac output stability over the measurement session (≈0.3%, based on the constancy of the reference readings), and charge measurement and leakage (≈0.1%). Combining these contributions in quadrature yields an estimated combined standard uncertainty of approximately 0.4% (*k* = 1) on the per‐configuration percent‐difference values. This uncertainty budget is considered when interpreting the practical significance of the measured differences in the Discussion.

### 2.2 Treatment planning comparison

The head phantom was scanned on a CT simulator using a clinical SRS head protocol with each mask system in place. Images were acquired at 120 kVp with a 1.0 mm slice thickness and reconstructed onto a 512 × 512 matrix using a standard reconstruction kernel, consistent with our clinical intracranial SRS simulation protocol. The active volume of the ionization chamber was contoured on each CT dataset, and planning target volumes (PTVs) were generated by expanding the detector contour with uniform margins of 1, 3, 5, 7, 10, and 15 mm, creating targets of varying size to evaluate the effect of target volume on dose calculation accuracy.

HyperArc treatment plans were generated in the Eclipse treatment planning system (version 16.1, Varian Medical Systems, Palo Alto, CA) for each PTV. Plans were optimized using default SRS parameters with an SRS normal tissue objective (NTO) of 100 and automatic lower dose objective. Each plan was calculated using two dose calculation algorithms, AAA and AXB (version 16.1), with a 1.0 mm (1.25 mm for AAA) calculation grid size. Acuros XB was configured to report dose‐to‐medium (Dm), consistent with our clinical SRS practice.

For each mask system and algorithm combination, plans were calculated under two body contouring conditions: (1) the default body contour, which did not include the mask material, and (2) a modified body contour that encompassed the mask. The mask contour was generated using a fixed Hounsfield‐unit threshold to capture the mask shell, followed by manual review and editing on every slice to ensure the contour conformed to the mask material. The encompassed mask volume was assigned its native CT Hounsfield units (i.e., no density override was applied).

The TPS‐calculated mean dose to the detector active volume contour served as the predicted dose. Plans were delivered on the linear accelerator, and the measured ion chamber reading was converted to dose using the cross‐calibration factor. Dose calculation accuracy was expressed as the percent difference between TPS‐predicted and measured dose for each condition.

To evaluate clinical relevance for multi‐target SRS, an additional set of HyperArc plans was created for both mask systems using three PTVs simultaneously (simulating a multi‐metastasis treatment) with margins of 3, 5, and 7 mm. These plans were calculated under the same four conditions (AAA and AXB, with and without mask contouring) and verified against ion chamber measurements using the same methodology described above.

### 2.3 Patient positioning evaluation

A retrospective analysis of patient setup correction data was performed for SRS treatments delivered at our institution using each mask system. Setup corrections were recorded from the image‐guided radiation therapy (IGRT) workflow using cone‐beam computed tomography (CBCT) alignment, including six degrees of freedom: three translational shifts (vertical, longitudinal, lateral) and three rotational corrections (rotation, roll, pitch).

The Encompass dataset comprised 67 fractions from 21 patients treated between January and April 2026, whereas the Environ dataset comprised 72 fractions from 24 patients treated during the same period. The same CBCT‐based 6‐DoF registration workflow, automatic registration algorithm, and clinical action thresholds were used throughout both periods, and no major modifications to the cranial SRS setup or imaging protocol were introduced during the transition between systems. For each degree of freedom, both signed mean shifts and absolute (unsigned) mean shifts were calculated, together with standard deviations.

Because multiple fractions from the same patient are not statistically independent, the positioning analysis was conducted at the patient level. For each patient, the mean correction across their fractions was computed for every degree of freedom, and between‐system differences in these patient‐mean corrections were assessed using Mann–Whitney *U* tests. Differences in patient‐to‐patient variability were evaluated using Levene's test applied to the patient‐mean corrections. To assess fraction‐to‐fraction reproducibility within patients, the standard deviation of corrections across each patient's fractions was computed, and the resulting within‐patient standard deviations were compared between systems using Mann–Whitney *U* tests. As a confirmatory analysis, a linear mixed‐effects model was fit for each degree of freedom with mask system as a fixed effect and patient as a random effect. Statistical significance was defined as *p* < 0.05. Patients receiving only a single fraction were included in the patient‐level mean and mixed‐effects analyses but excluded from the within‐patient reproducibility analysis, for which a within‐patient standard deviation is undefined.

## 3 RESULTS

### 3.1 Dosimetric absorption

#### 3.1.1 Static gantry measurements

Ion chamber readings for the seven static gantry beams are presented in Table 1. The Environ mask consistently produced higher chamber readings than the Encompass mask at every gantry angle, indicating less beam attenuation. Relative differences ranged from −0.33% (at 150°) to −1.82% (at 90°), with the largest differences observed at lateral beam angles where the beam path traverses the thickest portion of the mask material. This angular dependence is consistent with previous evaluations of thermoplastic mask attenuation.^5^, ^8^

**TABLE 1:** Static gantry ion chamber measurements for the Encompass and Environ mask systems. Negative differences indicate higher readings (less attenuation) with the Environ mask.

Gantry Angle	Encompass R1 (nC)	Encompass R2 (nC)	Encompass Avg (nC)	Environ R1 (nC)	Environ R2 (nC)	Environ Avg (nC)	Diff (%)
0°	5.74	5.74	5.74	5.79	5.79	5.79	−0.87
30°	5.71	5.71	5.71	5.78	5.78	5.78	−1.23
60°	6.31	6.31	6.31	6.41	6.41	6.41	−1.58
90°	6.03	6.03	6.03	6.14	6.14	6.14	−1.82
120°	6.43	6.43	6.43	6.53	6.53	6.53	−1.56
150°	6.01	6.01	6.01	6.03	6.03	6.03	−0.33
180°	6.07	6.07	6.07	6.10	6.10	6.10	−0.49

The mean relative difference across all static gantry angles was −1.13% (SD = 0.59%). A paired *t*‐test confirmed that this difference was statistically significant (*p* = 0.002), indicating that the Encompass mask attenuates measurably more radiation than the Environ mask under static beam conditions. The largest angle‐specific differences (up to −1.82% at 90°) exceed the estimated combined measurement uncertainty of approximately 0.4% (*k* = 1; Section 2.1) by more than a factor of four, supporting their physical significance. The smallest differences (−0.33% at 150° and −0.49% at 180°) are comparable to the measurement uncertainty and should be interpreted with corresponding caution.

#### 3.1.2 Arc delivery

Arc delivery results are summarized in Table 2. The difference between the two masks for the 0°–180° arc was −0.06%, well within the estimated combined measurement uncertainty of approximately 0.4% and therefore not distinguishable from zero. This is consistent with the expectation that rotational delivery averages angular attenuation differences.^8^

**TABLE 2:** Arc delivery ion chamber measurements.

Arc	Encompass R1	Encompass R2	Encompass Avg	Environ R1	Environ R2	Environ Avg	Diff (%)
0°–180°	48.83	48.83	48.83	48.86	48.86	48.86	−0.06

### 3.2 Treatment planning accuracy

#### 3.2.1 Single‐target encompass plans

Table 3 presents the percent difference between TPS‐calculated and measured dose for single‐target HyperArc plans with the Encompass mask. Without the mask included in the body contour, the AAA dose calculation was higher than measured dose by an average of 3.21% across all PTV sizes (range: 1.92%–7.91%), while the AXB dose calculation was higher by an average of 2.38% (range: 0.77%–3.39%). When the mask was contoured within the body structure, agreement improved substantially: AAA showed an average difference of 1.16% (range: −0.15%–5.61%), and AXB showed an average difference of 0.10% (range: −1.01%–1.61%). The improvement with mask contouring is consistent with the recommendations of AAPM TG‐176 to model immobilization devices in the TPS.^5^

**TABLE 3:** Percent difference between TPS‐calculated and measured dose for single‐target HyperArc plans with the Encompass mask.

Margin (mm)	AAA No Mask (%)	AAA Mask (%)	AXB No Mask (%)	AXB Mask (%)
1	7.91	5.61	3.24	1.61
3	2.81	0.72	3.39	1.20
5	1.92	−0.15	2.91	0.81
7	2.14	0.08	2.47	0.48
10	2.03	0.14	1.48	−0.30
15	2.43	0.58	0.77	−1.01

The 1 mm margin PTV consistently showed the largest discrepancy across all conditions, reflecting the high dose gradient at the edge of a very small target and the challenge of accurately computing dose to a volume approaching the detector's physical dimensions.

#### 3.2.2 Single‐target environ plans

Table 4 presents equivalent data for the Environ mask. Without mask contouring, AAA produced a mean difference of 1.09% (range: −0.39%–5.94%), while AXB showed a mean difference of 0.24% (range: −0.90%–1.21%). With the mask contoured, AAA yielded a mean difference of −0.25% (range: −1.75%–4.48%), and AXB showed a mean difference of −1.04% (range: −2.07%–−0.22%). The Environ mask plans demonstrated overall smaller absolute differences without mask contouring compared to the Encompass mask, particularly with the AXB algorithm, consistent with the reduced attenuation observed in the static beam measurements.

**TABLE 4:** Percent difference between TPS‐calculated and measured dose for single‐target HyperArc plans with the Environ mask.

Margin (mm)	AAA No Mask (%)	AAA Mask (%)	AXB No Mask (%)	AXB Mask (%)
1	5.94	4.48	1.21	−0.22
3	0.46	−0.97	0.28	−1.03
5	−0.39	−1.75	0.65	−0.63
7	−0.39	−1.71	0.01	−1.27
10	0.36	−0.86	0.18	−1.02
15	0.55	−0.68	−0.90	−2.07

**FIGURE 2:**
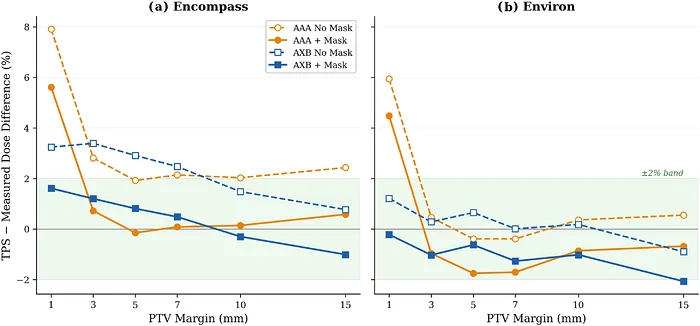
Percent difference between TPS‐calculated and measured dose as a function of PTV margin for the Encompass (left) and Environ (right) mask systems. Four conditions are shown: AAA without mask contouring (orange dashed, open circles), AAA with mask contouring (orange solid, filled circles), AXB without mask contouring (blue dashed, open squares), and AXB with mask contouring (blue solid, filled squares). The green shaded region indicates the ± 2% agreement band.

The effect of algorithm and mask contouring across all PTV margin sizes is illustrated in Figure 2 for both mask systems. For the Encompass mask (left panel), AXB with mask contouring (blue solid line with filled squares) yields the closest agreement with measurement across all margin sizes except 1 mm. For the Environ mask (right panel), the no‐mask conditions already fall within the ± 2% band for most margins. Adding mask contouring to the Environ AXB calculation introduces a small negative bias at most margins, although the effect is not uniform: agreement improves at the 1 mm margin (from +1.21% to −0.22%) and is essentially unchanged at 5 mm (+0.65% to −0.63%), while the remaining margins shift further below zero.

#### 3.2.3 Multi‐metastasis planning

Table 5 presents results for the multi‐metastasis HyperArc plans using both mask systems with PTVs at 3, 5, and 7 mm margins. For the Encompass mask, AAA without mask contouring averaged 4.04% difference to measured dose and AXB without mask contouring averaged 2.33%; with mask contouring these improved to 2.10% and 1.70%, respectively. For the Environ mask, AAA without mask contouring averaged 3.03% difference to measured dose and AXB without mask contouring averaged 1.71%; with mask contouring these improved to 0.76% and −0.25%, respectively.

**TABLE 5:** Percent difference between TPS‐calculated and measured dose for multi‐metastasis HyperArc plans comparing the Encompass and Environ mask systems.

Margin (mm)	AAA No Mask (%)	AAA Mask (%)	AXB No Mask (%)	AXB Mask (%)
**Encompass**				
3	6.73	4.62	2.60	0.62
5	4.77	2.78	3.92	2.29
7	0.62	−1.09	0.48	2.20
**Avg**	**4.04**	**2.10**	**2.33**	**1.70**
**Environ**				
3	5.64	2.25	4.24	0.95
5	3.21	0.87	1.50	0.08
7	0.25	−0.83	−0.60	−1.79
**Avg**	**3.03**	**0.76**	**1.71**	**−0.25**

The multi‐met results follow the same pattern observed in the single‐target data: the Environ mask produced smaller baseline discrepancies than the Encompass mask across all four calculation conditions, and mask contouring improved agreement for both systems. At the 7 mm margin the two systems deviated in opposite directions, with the Encompass AXB‐with‐mask plan yielding +2.20% and the Environ AXB‐with‐mask plan yielding −1.79%, whereas at the 3 and 5 mm margins both systems deviated in the same (positive) direction. The largest AXB‐with‐mask deviation occurred at 5 mm for the Encompass (+2.29%) and at 7 mm for the Environ. The opposing signs of the 7 mm deviations, occurring for the same plan geometry across both mask systems, indicate that this behavior is not attributable to a mask‐specific property but rather to the multi‐target noncoplanar arc geometry at that particular target size and location.

### 3.3 Patient positioning

Table 6 summarizes the patient‐level setup correction data for both mask systems. Mean translational shifts were small for both systems, with the vertical direction showing the largest mean corrections (Encompass: 0.86 ± 1.00 mm; Environ: 0.29 ± 0.59 mm, expressed as the mean ± standard deviation of patient‐mean corrections). Longitudinal and lateral mean shifts were below 0.15 mm for both systems. Mean rotational corrections were below 1° for all axes in both systems, with pitch showing the largest values (Encompass: −0.68 ± 0.84°; Environ: −0.29 ± 0.64°). Absolute (unsigned) mean corrections are reported alongside signed values in Table 6.

**TABLE 6:** Patient‐level setup correction statistics for the Encompass (21 patients) and Environ (24 patients) mask systems. All values are derived from patient‐level summaries rather than pooled fractions. Mean patient correction (signed) and Mean patient correction (absolute) are the mean across patient‐mean corrections; between‐patient SD is the standard deviation of patient‐mean corrections; within‐patient SD is the mean of each patient's fraction‐to‐fraction standard deviation. Translational values in millimeters, rotational values in degrees.

Parameter	Vertical (mm)	Long. (mm)	Lateral (mm)	Rotation (°)	Roll (°)	Pitch (°)
**Encompass — Mean patient correction (signed)**	0.86	−0.07	0.02	−0.09	−0.08	−0.68
Encompass — Mean patient correction (absolute)	1.40	0.19	0.15	0.74	0.65	1.14
Encompass — Between‐patient SD	1.00	0.15	0.20	0.62	0.62	0.84
Encompass — Within‐patient SD	1.25	0.19	0.16	0.77	0.58	0.99
**Environ — Mean patient correction (signed)**	0.29	−0.10	0.00	−0.14	0.03	−0.29
Environ — Mean patient correction (absolute)	0.47	0.27	0.13	0.65	0.58	0.67
Environ — Between‐patient SD	0.59	0.25	0.13	0.55	0.47	0.64
Environ — Within‐patient SD	0.31	0.27	0.13	0.57	0.55	0.50

At the patient level, the Mann–Whitney *U* test indicated a significant between‐system difference in mean vertical shift (*p* = 0.035); differences in the remaining five degrees of freedom were not statistically significant (p ≥ 0.099). Levene's test on patient‐mean corrections indicated significantly lower patient‐to‐patient variability for the Environ system in the vertical direction (*p* = 0.016); no other axis reached significance. Comparison of within‐patient (fraction‐to‐fraction) standard deviations showed significantly smaller values for the Environ system in the vertical (mean 0.31 vs. 1.25 mm, *p* < 0.001) and pitch (mean 0.50 vs. 0.99°, *p* = 0.009) directions; the remaining axes did not differ significantly. The mixed‐effects models, with patient as a random effect, were consistent with the patient‐level tests: the vertical system effect was significant (*p* = 0.016) while the pitch system effect was not (*p* = 0.080).

**FIGURE 3:**
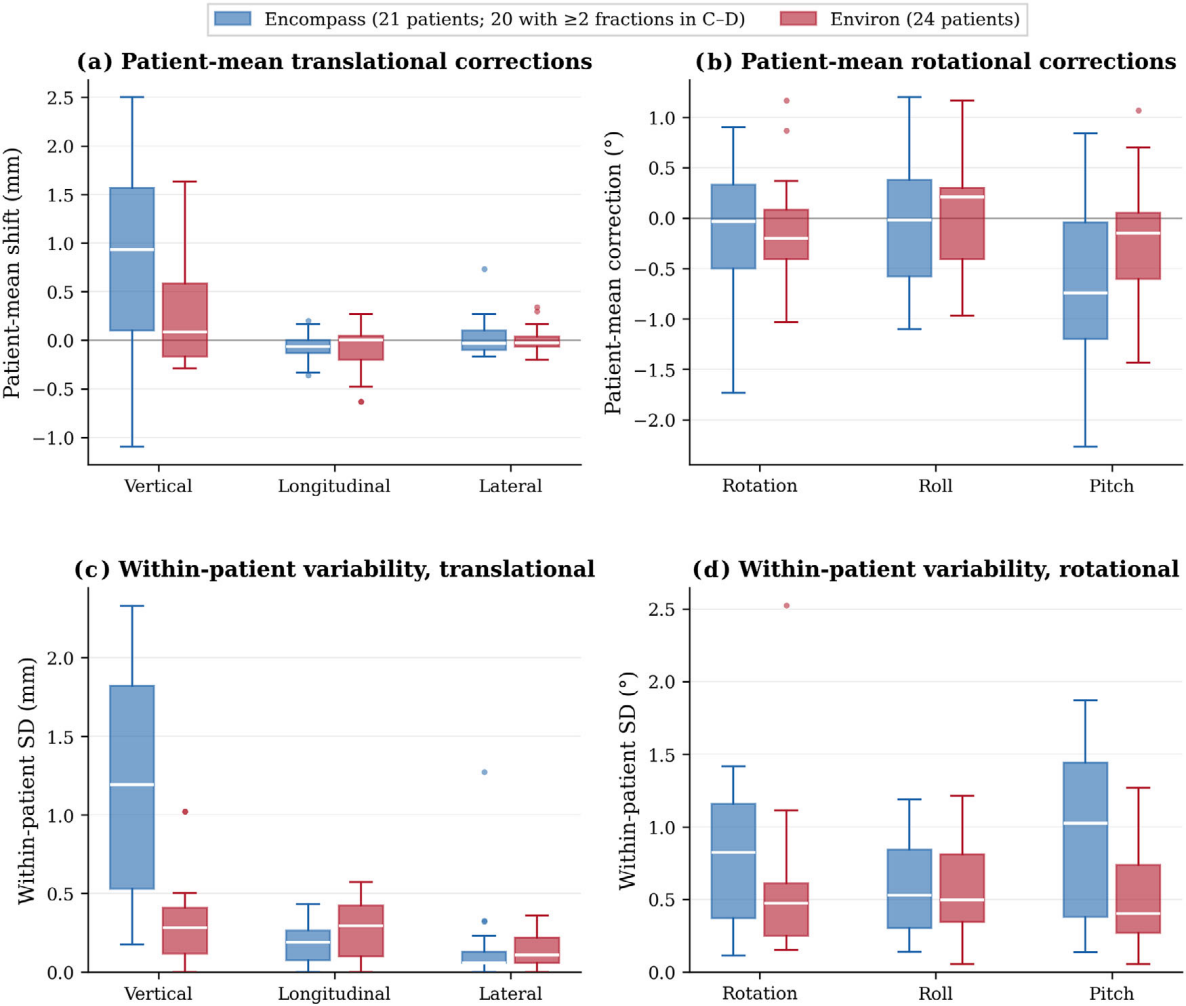
Patient‐level setup correction distributions for the Encompass (blue, 21 patients) and Environ (red, 24 patients) mask systems. (a) Patient‐mean translational corrections in millimeters. (b) Patient‐mean rotational corrections in degrees. (c, d) Within‐patient (fraction‐to‐fraction) standard deviations for the translational and rotational axes, respectively. Each observation represents one patient. Panels C and D include the 20 Encompass patients with at least two fractions, as a within‐patient standard deviation is undefined for single‐fraction patients. Boxes represent the interquartile range, whiskers extend to 1.5× IQR, and individual points indicate outliers.

The patient‐level distributions underlying these comparisons are illustrated in Figure 3, which presents patient‐mean corrections (panels A and B) and within‐patient standard deviations (panels C and D) for each degree of freedom, matching the units of analysis used for the statistical tests reported above.

## 4 DISCUSSION

This study presents a comprehensive three‐part evaluation of the Environ SRS immobilization mask system compared to the established Encompass HyperArc mask at our institution. The results provide quantitative evidence across three performance domains relevant to clinical implementation: beam attenuation, dose calculation accuracy, and interfraction setup reproducibility.

### 4.1 Dosimetric absorption

The static gantry measurements revealed that the Encompass mask attenuates approximately 1.1% more dose on average than the Environ mask. While statistically significant, this magnitude is generally within the range considered clinically acceptable for SRS immobilization devices.^5^, ^8^ The angle‐dependent variation in attenuation (0.33%–1.82%) reflects differences in the mask material thickness and geometry encountered at different beam entry angles, with lateral beams (60°–90°) traversing more material and showing the largest differences. Snyder et al. similarly observed that attenuation through the Encompass system was highest at lateral angles where beams pass through the mask attachment regions.^8^ Ferrer et al. reported comparable angular attenuation patterns for the Fraxion immobilization system, with differences of up to 2% at oblique angles.^16^

The negligible difference observed for arc delivery (−0.06%) is expected, as the continuous gantry rotation averages the attenuation across all angles, effectively minimizing the impact of angle‐dependent thickness variations.^8^ This finding is reassuring for clinical HyperArc treatments, which primarily use non‐coplanar arc delivery,^6^, ^7^ and suggests that for arc‐based SRS the choice between these two masks is unlikely to produce clinically meaningful attenuation differences.

### 4.2 Treatment planning accuracy

The treatment planning comparison yielded several important findings with direct clinical implications. Including the mask material in the body contour generally improved agreement for the Encompass system and for AAA‐based calculations. For the Environ system calculated with AXB, however, the no‐mask condition already demonstrated close agreement with measurement, and explicit mask inclusion shifted the results systematically negatively, suggesting mild overcorrection under the tested CT‐to‐density assignment.

Second, the AXB algorithm demonstrated superior accuracy compared to AAA across nearly all conditions, as illustrated in Figure 2. This advantage is consistent with the known physics of the AXB algorithm, which solves the linear Boltzmann transport equation and more accurately models heterogeneity corrections.^12^, ^13^ Fogliata et al. demonstrated that AXB provides improved small‐field dose calculation accuracy compared to AAA, with particular relevance for stereotactic treatments.^17^ Menon et al. further validated this finding specifically for RapidArc‐based SRS plans for brain metastases, showing AXB provides more accurate in‐field dose estimation.^14^

Third, the interaction between mask contouring and algorithm choice is noteworthy. For the Environ mask with AXB, the no‐mask condition already achieved a mean difference of only 0.24%, while adding mask contouring shifted the result to −1.04%. This behavior may reflect slight over‐attenuation of the Environ mask material by the TPS when it is explicitly modeled, which could be related to the CT‐number‐to‐density conversion for the mask material or to the thinner profile of the Environ mask. Snyder et al. investigated optimal HU assignments for the Encompass TPS model and found that values of 400 and −400 for the Encompass structure and base structure, respectively, provided best agreement with measurements.^8^ Similar optimization may be warranted for the Environ mask.

These findings support different optimal planning workflows for the two mask systems. For the Encompass, mask contouring combined with AXB calculation provided the best agreement and appears necessary under the investigated conditions to achieve clinically acceptable results—without mask contouring, even AXB produced mean deviations of 2.38% for single targets and 2.33% for multi‐met plans. For the Environ, by contrast, AXB calculation alone achieved sub‐1% mean agreement for single targets (0.24%) and 1.71% for multi‐met plans even without mask contouring, while adding mask contouring introduced a small negative bias. This represents a potential practical advantage for the Environ system: under the conditions tested, accurate dose calculation could be achieved with the default body contour, eliminating an additional contouring step and a potential source of user variability in the planning workflow. Working tolerances reported for microchamber‐based SRS end‐to‐end testing are on the order of 5% for AAA and 2% for AXB, and the Environ met the AXB tolerance under both planning workflows.

The multi‐metastasis planning results reinforced the trends observed for single‐target plans. Both mask systems showed improved agreement with mask contouring, and the Environ mask again produced smaller baseline discrepancies than the Encompass mask in all four conditions. At the 7 mm margin the two systems deviated in opposite directions (Encompass AXB‐with‐mask: +2.20%; Environ AXB‐with‐mask: −1.79%). Because the sign of the deviation would be expected to be consistent across systems if mask material were the driver, this pattern is unlikely to be attributable to a property of either immobilization system. It may instead reflect plan‐specific effects, which could be related to interactions between the multi‐target noncoplanar arc geometry, MLC modulation for the larger off‐axis target, and microchamber sampling at the edge of the PTV; the present data do not allow these contributions to be separated. These findings underscore the importance of end‐to‐end verification of multi‐target SRS plans on a case‐by‐case basis, regardless of the mask system used, as has been emphasized in reports on SIMT dosimetry.^18^

### 4.3 Patient positioning

The CBCT setup‐correction data showed that both mask systems achieve sub‐millimeter mean translational corrections and sub‐degree mean rotational corrections, consistent with published expectations for open‐face thermoplastic SRS masks used with CBCT‐based IGRT.^3^, ^4^, ^9^ It is important to note that these data reflect interfraction setup reproducibility derived from pre‐treatment CBCT registration, and not intrafraction motion or overall immobilization performance. Assessment of intrafraction stability would require post‐treatment imaging, intrafraction CBCT, or continuous surface‐guided monitoring, none of which were available in this retrospective dataset. Indeed, in surface‐guided workflows the principal role of the open‐face mask is to permit reliable monitoring rather than to fully immobilize the patient,^10^ underscoring that setup reproducibility and immobilization performance are distinct endpoints. Babic et al. compared multiple frameless immobilization systems and reported mean intrafraction motion of 0.73–0.76 mm for thermoplastic masks^3^; our interfraction setup corrections are of comparable magnitude but address a different stage of the treatment chain.

The vertical direction showed the largest setup corrections for both systems. Previous studies of thermoplastic mask fixation have attributed this pattern to the anterior‐posterior axis being particularly susceptible to mask settling and deformation^3^, ^19^; the present study did not directly assess mask deformation, and this mechanism is therefore offered as a possible explanation drawn from the literature rather than a finding of this work. After accounting for within‐patient clustering, the Environ system showed a significantly smaller mean vertical correction (patient‐level Mann–Whitney *p* = 0.035; mixed‐effects *p* = 0.016) and significantly reduced within‐patient variability in the vertical (*p* < 0.001) and pitch (*p* = 0.009) directions. The reduction in within‐patient (fraction‐to‐fraction) variability is the finding most relevant to setup reproducibility, as it reflects the consistency with which a given patient is re‐positioned across fractions. The remaining degrees of freedom did not differ significantly between systems. These results suggest improved vertical and pitch setup reproducibility with the Environ system, although the magnitude of the mean differences (on the order of 0.4 mm and 0.4°) is small relative to typical SRS PTV margins. A potential confounder is that the two cohorts were treated during different time periods; although the CBCT registration workflow and action thresholds were unchanged, we cannot exclude that incremental gains in therapist experience and institutional familiarity contributed to the observed reduction in variability, rather than mask design alone. Lamba et al. reported similar magnitude setup corrections for frameless SRS and emphasized the importance of CBCT verification,^9^ and Tomihara et al. demonstrated that thermoplastic mask systems can achieve geometric accuracy sufficient for 1 mm PTV margins when combined with CBCT image guidance.^4^ Contemporary surface‐guided SRS series similarly report submillimeter setup accuracy with open‐face masks,^10^ reinforcing that both systems evaluated here perform within the clinically accepted range.

### 4.4 Limitations

The patient positioning analysis has several limitations. It is retrospective and compares non‐matched patient cohorts treated during different clinical periods; although the same CBCT registration workflow, automatic registration algorithm, and action thresholds were used throughout and no major protocol changes accompanied the transition, maturation of institutional experience over time cannot be fully excluded as a contributor to the observed differences in variability. The analysis evaluates only interfraction setup corrections from pre‐treatment CBCT and therefore does not characterize intrafraction motion or overall immobilization performance, which would require post‐treatment or continuous (e.g., surface‐guided) monitoring. Although the statistical analysis was performed at the patient level and confirmed with a mixed‐effects model to account for the non‐independence of repeated fractions, the number of patients per system remains modest and the study was not powered for the secondary degrees of freedom. Finally, fraction counts per patient were not identical between cohorts (median 3 for both; range 1–5 for Encompass, 3 for Environ), which the within‐patient reproducibility analysis partially mitigates but does not eliminate. Prospective studies with matched cohorts and intrafraction monitoring would be required to confirm these findings as differences in immobilization performance.

## 5 CONCLUSIONS

This integrated evaluation indicates that the Environ mask system is a clinically acceptable alternative to the Encompass for frameless intracranial SRS, performing comparably or favorably across dosimetric absorption, treatment planning accuracy, and interfraction setup reproducibility.

Under the specific imaging, calibration, and contouring conditions tested, the Environ achieved acceptable AXB dose‐calculation agreement without requiring the mask to be contoured in the body structure, whereas the Encompass required mask contouring to reach comparable agreement. Because this behavior depends on the CT acquisition protocol, HU‐to‐density calibration, contouring methodology, and TPS configuration, the reduced need for mask contouring with the Environ should be verified locally before being relied upon, and is not assumed to generalize to all institutions.

For interfraction setup reproducibility, the Environ showed a statistically significant advantage that survived patient‐level and mixed‐effects analysis only in the vertical direction, with reduced within‐patient variability additionally seen in pitch; the remaining axes were equivalent. These positioning findings are hypothesis‐generating given the retrospective, non‐matched design and the absence of intrafraction data.

This work provides a simultaneous evaluation of physical, dosimetric, and clinical performance for a candidate SRS immobilization system, an approach that may serve as a template for assessing future devices. Prospective, matched studies incorporating intrafraction monitoring—for example via surface‐guided radiotherapy—are warranted to confirm the reproducibility signals observed here and to characterize true immobilization performance.

## AUTHOR CONTRIBUTIONS

**Adi Robinson and Matthew R. Caldwell**: Conceptualization; methodology; interpretation of results; and preparation of the manuscript. **Matthew R. Caldwell**: Performed the majority of the data collection, and Adi Robinson performed the primary data analysis. Both authors reviewed and approved the final manuscript.

## CONFLICT OF INTEREST STATEMENT

The authors declare no conflicts of interest.
